# Transoral Laser Microsurgery (TLM) for Glottic Cancer: Prospective Assessment of a New Pathology Workup Protocol

**DOI:** 10.3389/fsurg.2020.00056

**Published:** 2020-08-28

**Authors:** Jeroen Meulemans, Esther Hauben, Samuel Peeperkorn, Sandra Nuyts, Pierre Delaere, Vincent Vander Poorten

**Affiliations:** ^1^Otorhinolaryngology, Head and Neck Surgery, University Hospitals Leuven, Leuven, Belgium; ^2^Section Head and Neck Oncology, Department of Oncology, Katholieke Universiteit Leuven, Leuven, Belgium; ^3^Pathology, University Hospitals Leuven, Leuven, Belgium; ^4^Department of Pathology and Imaging, Katholieke Universiteit Leuven, Leuven, Belgium; ^5^Radiation Oncology, University Hospitals Leuven, Leuven, Belgium; ^6^Section Experimental Radiotherapy, Department of Oncology, Katholieke Universiteit Leuven, Leuven, Belgium

**Keywords:** glottic cancer, laryngeal cancer, margins, transoral laser microsurgery, pathologic examination

## Abstract

**Background/Purpose:** The value of margin status after TLM for glottic cancer is debatable, due to difficulties in specimen orientation and margin analysis. Purpose of this study was the prospective evaluation of feasibility of a new standardized technique of oriented fixation of the TLM specimen and identification of the added value on tissue processing and margin status reporting.

**Methods:** Patients with suspicious glottic lesions undergoing TLM were included. After resection, the specimen margins were inked in the OR using different colors. Subsequently, the specimens were fixed on a pig liver carrier and sent for further processing, accompanied with photographs of the larynx pre-TLM and of the mounted specimen. Feasibility was assessed by registration of duration of specimen preparation in the OR and the lab and by procedure-specific questionnaires. Objective evaluation included assessment of margin status and proportion of evaluable margins. Chi square tests were used to make comparisons of proportions.

**Results:** One hundred and four consecutive patients were included between May 2016 and September 2019. TLM was performed in a primary and salvage setting in 89.4 and 10.6% of patients, respectively. Mean duration of intraoperative specimen preparation was 5.1 min (SD 2.6 min). No difficulties in orientation nor fixation during intraoperative preparation were reported in 87.5 and 88.2%, respectively. Specimen orientation was judged by the pathologist as very adequate in 89.4%, with the accompanying photographs considered helpful for orientation and processing in 84.6%. Substantial difficulties in further lab processing and pathologic examination were identified in 17.7%. Deep margin evaluability was very high (98.0%) and significantly higher than the evaluability of superficial mucosal margins. Compared to our previous series published by our group (*n* = 142), deep margin evaluability significantly rose from 62.7 to 98.0% (*p* < 0.001) and true positive rate of the deep margins increased from 0 to 44.4% (*p* = 0.002).

**Discussion/Conclusion:** The new and standardized technique of oriented fixation of TLM specimens on a pig liver carrier proves feasible both in the OR and lab setting and results in high margin evaluability rates, especially for the deep margin, as well as a decreased rate of false positive deep margins when compared to a historical TLM cohort.

## Introduction

Nowadays, transoral laser microsurgery (TLM) has a well-established role in the primary treatment of early (cTis-cT2) and well-selected cT3 glottic cancers, combining a high probability of local control with excellent laryngeal preservation rates (1–7). In well-selected cases, TLM can also be considered as a possible salvage treatment for radiorecurrent laryngeal cancers (8). As TLM can be considered a minimally invasive surgical technique aiming at leaving as much healthy tissue as possible untouched, it is characterized by the concept of tumor adapted resection with implementation of ultra-narrow margins (usually 1–3 mm). Using a combination of microscopic and endoscopic view, narrow superficial margins are defined and subsequently the tumor is most frequently transected to reveal the depth of tumor invasion and to determine the optimal deep tumor margin. As a result, the tumor is removed piecemeal through the laryngoscope. However, this concept of piecemeal resection implies significant difficulties in specimen orientation and margin analysis by the pathologist. Additionally, post-resection and post-fixation shrinkage of the resection specimen and thermal artifacts on the edges of the specimen caused by the carbon dioxide laser further hamper margin evaluation and are believed to be, in combination with the intentional narrow margin resection, partially responsible for the high rate of apparently unsafe margins after TLM with reported close and positive margin rates as high as 50% (3, 9). As a result of these problems the pathologist is faced with during pathological examination, the clinical value of margin status after TLM for glottic cancer is an ongoing matter of debate, with up to 80% of close and positive margins on definitive pathology believed to be false positive (10). Until now, the proposed solutions for enhanced precision and reliability of margin analysis are not always feasible from a practical point of view (e.g., orientation of the specimen by the surgeon in the pathology lab) or poorly standardized and poorly reproducible (e.g., pinning the specimen on custom made cardboard), resulting in difficult to interpret pathology reports. Together with the reported high rate of false positive and non-evaluable margins, decision-making on eventually needed adjuvant therapy after TLM (e.g., second-look TLM procedure, radiotherapy) and/or follow-up intensity is complicated and results in high rates of second look procedures. Intraoperative frozen section analysis of the margins has been suggested as a possible adjunct to limit the need for additional procedures, but its real value is controversial (4, 11). In this report, we prospectively evaluated feasibility of a newly in-house developed and standardized technique of oriented fixation of the surgical specimen on pig-liver slices in an intraoperative setting and aimed at identifying its potential added value on tissue processing and margin status reporting.

## Patients and Methods

### Study Outline

A prospective study was conducted at an academic tertiary referral hospital (University Hospitals Leuven, Leuven, Belgium) between May 2016 and September 2019. This study was approved by and carried out in accordance with the recommendations of the Institutional Review Board (University Hospital Leuven Committee for Medical Ethics, study number: S58892). Informed consent was obtained for every patient included in the study. All patients with glottic lesions suspect for malignancy who were scheduled for TLM resection of the lesion were eligible for inclusion, including both primary TLM cases as well as salvage TLM cases for radiorecurrent glottic cancer or for second primary glottic lesions in a previously irradiated larynx. Immediately after resection, the specimens were accurately inked in the operating theater under surgical loupe magnification using different colors to identify the different margins. After coloring margins, the specimens were fixed with cyanoacrylate glue on a pig liver carrier, photographed, and stored in formaldehyde. They then were sent for further processing to the pathology lab. The specimen was accompanied by digital photographs of the larynx with the tumor *in situ* before resection, taken via the operating microscope or endoscope, and of the mounted specimen. On both photographs, the inked margins were indicated by analogous coloring, as well as areas of specific interest. Feasibility was assessed by registration of duration of specimen preparation in the OR and the lab and by structured questionnaires assessing each step of the procedure and subjective physician satisfaction. Objective evaluation of the value of this technique included assessment of margin status and determination of the proportion of non-evaluable margins. Since this is a feasibility study, most outcomes are descriptive in nature.

### Surgery

**Figure 1:**
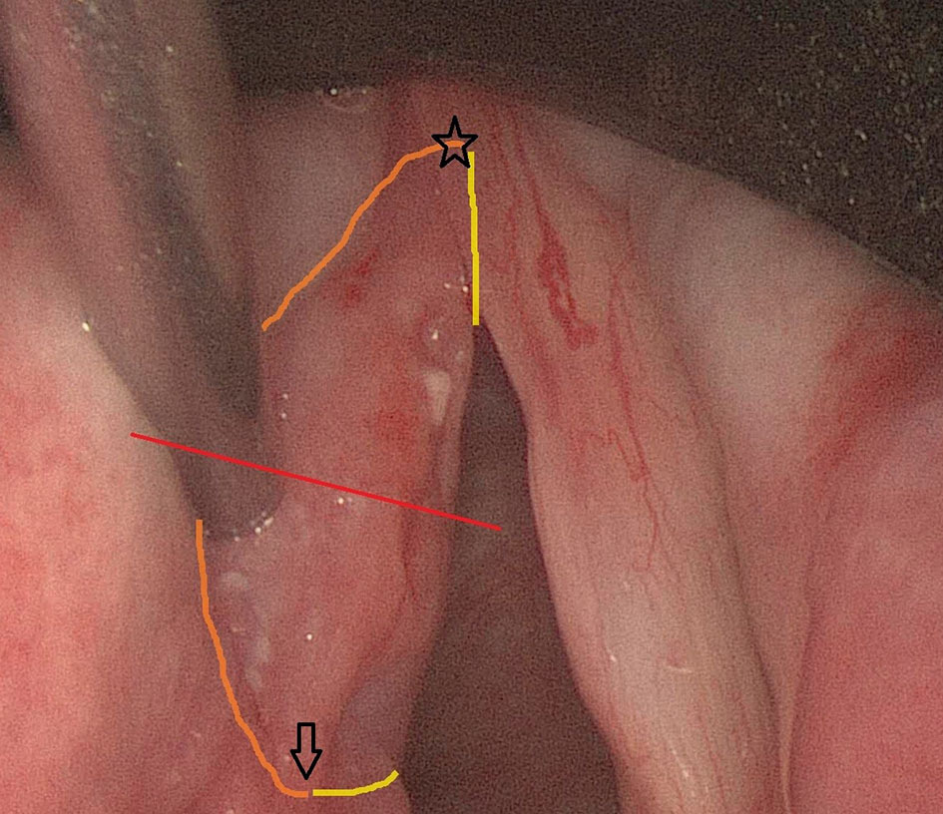
Peroperative endoscopic (0°) view on a cT1a SCC of the left true vocal fold prior to cordectomy type III. The red line indicates the line where deliberate tumor transection will be performed in order to evaluate depth of tumoral infiltration in the vocal fold. The anterior commissure, which also reflects the anterior tip of the specimen, is indicated by a star. The arrow represents the eventual posterior tip of the specimen. The orange contouring line depicts the lateral (cranial) margin, while the yellow line represents the medial (caudal) margin (only partially visible because of subglottic extension of the margin).

**Figure 2:**
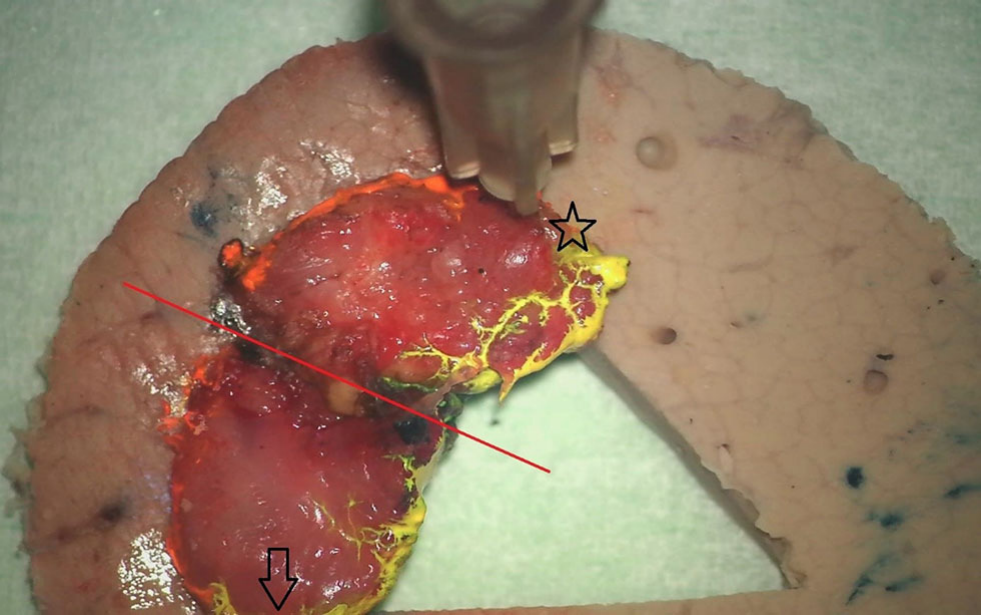
View on the oriented and inked specimen fixed on the underlying pig-liver carrier. The red line indicates the deliberate line of tumor transection and does not warrant any attention of the pathologist. Anterior and posterior tips of the specimen are marked with a star and arrow, respectively. Note the orange and yellow inking of the lateral (cranial) and medial (caudal) margins.

All TLM procedures were performed by the same surgeons (VV and JM). Patients were under general anesthesia and ventilated using a small diameter endotracheal tube (5.5 or 6 mm), high-frequency jet-ventilation (HFJV) or Evone® flow-controlled ventilation (12). After optimal exposure of the glottic area using different closed laryngoscopes (Karl Storz, Tüttlingen, Germany), the extent and location of the tumor was assessed using 0, 30, and 70° endoscopes and detailed pictures of the tumor site were taken and stored in the patient's electronic medical file. A CO_2_-laser (AcuPulse Duo, Lumenis, Israël) equipped with a micromanipulator attached to the operating microscope (OPMI Vario, Zeiss, Göttingen, Germany) was used for all TLM procedures, which were classified as recommended by the European Laryngological Society (13, 14). Most frequently, a piecemeal resection was achieved after cutting through the tumor, allowing for an intraoperative exploration of depth of invasion and determination of a safe deep margin. Only the smallest and most superficial glottic lesions were removed en bloc. After resection, the deep margins of the specimens were accurately inked under magnification using surgical loupes with blue ink (Davidson Marking system Refill bottles of 59 ml) applied on a 22G needle. The ink on the deep margin was fixed with AFA/formaldehyde spray and subsequently, the specimens were fixed with cyanoacrylate glue (LOCTITE 401 3 gr Universal, 0.5 ml aspirated in a 1 ml Insulin Syringe and using a 28 G needle for glue application) on a pig liver carrier. These carriers were prepared by the pathology lab as a circular slice of 3 cm diameter with excised triangle in the center, mimicking a horizontal cross-section of the glottic larynx and stored in 4% buffered formaldehyde, but dried with a cotton before fixation of the specimen. An interesting property of this carrier is that it can be lamellated together with the mounted specimen in the microtome. After fixation, lateral (cranial) margins were inked orange and medial (caudal) margins yellow. After completion of specimen preparation, digital photographs of the inked specimen-carrier were taken whereupon the complex was stored in 4% buffered formaldehyde solution. These images were printed out together with the images of the preoperative tumor site and both printouts were provided with written analogous orientation markings (cranial, caudal, anterior, posterior). The inked margins of the resection specimen were indicated on the image of the preoperative tumor site by analogous coloring and areas of specific interest for the pathologist (e.g., plane of deliberate tumor transection, margins judged by the surgeon as possibly compromised during TLM) were indicated on both images. Eventually, the specimen-carrier complex was sent to the pathology lab accompanied by the printed and adapted images. Figures 1, 2 are examples of images of the tumor site and the specimen-carrier complex with analogous markings. A second look TLM procedure 6–8 weeks after the first surgery was preferentially scheduled when definitive pathologic examination suggested a deep margin positive for invasive SCC. In cases with multiple superficial margins positive for invasive SCC or CIS, a second look TLM was performed when the treating surgeon experienced intra-operative doubts about radicality.

### Pathologic Examination

The resected specimen, together with the pig liver carrier, was lamellated from anterior to posterior in slices of 3 mm thickness. The whole specimen was placed in pathology specimen cassettes for further processing to paraffin blocks. The anterior, posterior and central slices were embedded in separate cassettes. The material was routinely processed and H&E stained sections were made. Whenever high grade dysplasia, carcinoma *in situ* or invasive carcinoma was seen during microscopy in the anterior or posterior tip of the specimen, the paraffin block was melted down, and the specimen was embedded with the other side of the slice up. New slides were then made with minimal trim in order to evaluate the extreme margin of the specimen.

### Data and Statistics

Data were entered into an electronic case report file (eCRF) (Access 2016, Microsoft, Redmond, USA).

Objective data registered during TLM and during preparation of the specimen in the operating room (OR) were: date of diagnosis, date of TLM, tumor side, primary tumor location, extension of the tumor, cT classification, primary or salvage setting, duration of TLM procedure (in minutes, as measured preoperatively using a stopwatch), ELS type of cordectomy, number of resected pieces (in case of a piecemeal resection), and processing time in operating theater (time needed for orienting, inking and fixation of the specimen(s), time needed to produce photographs and marked printouts as measured in seconds). Subjective data registered related to the ease of specimen processing and surgeon satisfaction. Procedure-specific questionnaires assessed difficulties during specimen orientation and specimen fixation, on a scale from 1 (no difficulties at all) to 4 (very much). If applicable, experienced difficulties were specified in the eCRF.

Objective data registered in the pathology lab during specimen processing and evaluation were: time from start of the preparation process by the lab technician to finalization with the specimen ready for slicing (in minutes, as measured using a stopwatch), worst histology [invasive squamous cell carcinoma (SCC), *in situ* carcinoma (CIS), high grade dysplasia, low grade dysplasia], status of the deep, cranial (lateral), caudal (medial) margins and of the anterior and posterior specimen tips (close, positive, free, or non-evaluable). If margins or tips were judged close or positive, the histology of the transected tissue was specified (invasive SCC, CIS, or dysplasia). A free margin was defined as a margin of ≥1 mm. Subjective data registered related to the ease of specimen processing and pathologist satisfaction. Procedure-specific questionnaires assessed difficulties during specimen orientation and workup on a scale from 1 (no difficulties at all) to 4 (very much). If applicable, difficulties were specified in the eCRF. The added value of the accompanying marked photographs was scored on a scale from 1 (not helpful) to 4 (very helpful).

Moreover, the rate of second look TLM procedures and pathology of second look specimens (negative, dysplasia, CIS, or invasive SCC) were registered. A “true positive margin” was defined as a positive margin (deep or superficial) on initial pathologic examination which was confirmed as positive at second look resection.

Data were analyzed using SPSS version 22.0 statistical software (IBM corp, Armonk, NY, USA). Deep margin evaluability rates resulting from the new pathology workup were compared to observed rates in a previously published TLM cohort using standard orientation (e.g., cardboard) (15). Superficial margin evaluability rates could not be compared due to lacking data on superficial margin status in the historical cohort. Univariable comparison of both cohorts was performed using χ^2^ test (cT distribution and primary vs. salvage setting; 5% significance level). Fisher's exact test was used to compare “deep margin true positive rates” between the historic and current TLM cohorts, and to compare “true positive rates” of the deep margins and superficial margins.

## Results

### Patient, Tumor, and Treatment Characteristics

We included 104 patients in this prospective feasibility study. Tumors were pre-operatively staged as cTis (*n* = 1; 1.0%), cT1a (*n* = 68; 65.4%), cT1b (*n* = 14; 13.5%), cT2 (*n* = 20; 19.2%), and cT3 (*n* = 1; 1%). All patients were cN0. Ninety-three patients (89.4%) underwent primary TLM, 11 patients (10.6%) were treated in a salvage setting. Type of TLM procedures performed were cordectomy type I (*n* = 26; 25.0%), II (*n* = 25; 24.0%), III (*n* = 30; 28.8%), Va (*n* = 15; 14.4%), Vb (*n* = 3; 2.9%), Vd (*n* = 2; 1.9%), and VI (*n* = 3; 2.9%). In exactly half of cases (*n* = 52; 50%), the tumor was removed en bloc, while piecemeal resection was performed for the remaining half (two pieces in 43 patients or 41.3%, three pieces in seven patients or 6.7%). Mean TLM duration was 30.6 min (range 3–150 min, SD 28.2 min).

### Feasibility Results

Mean duration of the specimen preparation in the OR, including inking of margins, fixing on the pig-liver carrier and producing annotated photographs, was 5.1 min (range 1.0–30.0 min, SD 2.6 min). Subjectively, no difficulties in orientation nor fixation were reported in 87.5 and 88.2% of patients, respectively.

In the pathology lab, the mean time needed to prepare the specimen-carrier complex for slicing was 8.9 min (range 2–32 min, SD 6.1 min). During evaluation, no difficulties with specimen orientation (score 1) were encountered in 89.4% of specimens. The accompanying photographs were judged helpful (scores 2–3–4) during orientation and further processing in 84.6% of specimens. Substantial difficulties (score 3) were reported in 17.7% of specimens, the most common problems being the presence of laser coagulation artifacts hampering margin evaluation (*n* = 12) and the specimen loosening from the pig liver carrier during processing (*n* = 10).

### Pathology Results and Margin Analysis

**Figure 3:**
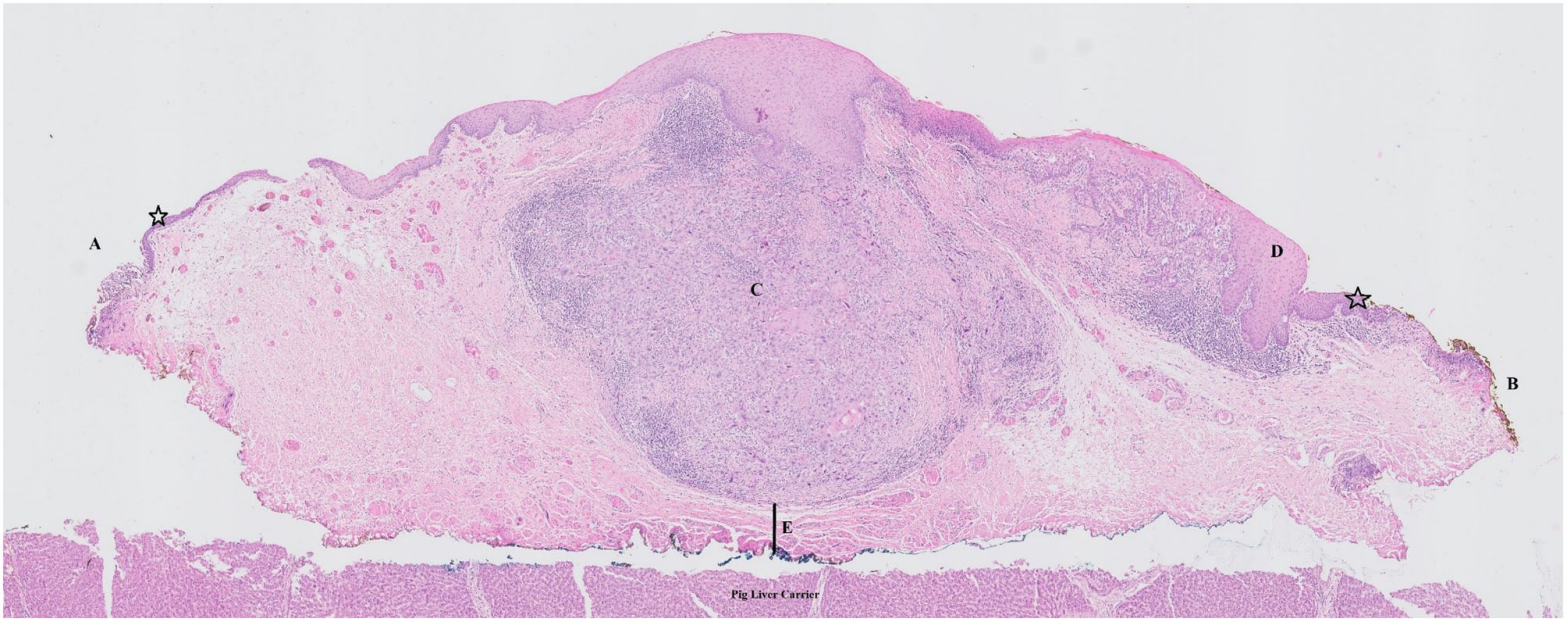
Microscopic view of a transverse section of a TLM specimen—pig liver carrier complex. Note the excellent evaluability of the deep margin. Stars indicate normal epithelial layer. **(A)** free superficial cranial margin; **(B)** close superficial caudal margin; **(C)** zone of squamous cell carcinoma with invasion of the submucosa/vocal ligament but without invasion in the underlying vocalis muscle; **(D)** zone of epithelial dysplasia; **(E)** close deep margin.

The worst histology encountered in the specimen during pathologic evaluation was low- and high-grade dysplasia in 9 (8.7%) and 10 (9.6%) cases, respectively, carcinoma *in situ* in 21 (20.2%) cases and invasive SCC in 62 (59.6%) specimens. During evaluation, evaluability and status (free, close, positive) of all margins (deep, cranial, caudal, posterior tip, and anterior tip) were assessed and registered (Table 1). Deep margin evaluability rate proved very high (98.0%) and significantly higher than the evaluability rate of superficial mucosal margins [86.1% for cranial margin (*p* = 0.002), 87.1% for caudal margin (*p* = 0.004), 81.0% for the anterior tip (*p* < 0.001), and 80.2% for the posterior tip (*p* < 0.001)]. The deep margin was free or close in 85.9% of specimens, compared to a free/close margin rate of 57.7% for the cranial margin (*p* = 0.002), 64.4% for the caudal margin (*p* = 0.041), 38% for the anterior tip (*p* < 0.001) and 35.6% for the posterior tip (*p* < 0.001). Multiple superficial margin positivity (for CIS and/or invasive SCC) was observed in 22.8% of specimens. We did not find a significant difference in deep margin status or evaluability between the primary and the salvage TLM group: 56.9 vs. 72.7% free margins, 20.5 vs. 9.1% close margins, 11.4 vs. 18.2% positive margins and 2.3 vs. 0% non-evaluable margins, respectively (*p* = 0.717). Specifications of the histology (invasive SCC, CIS, dysplasia) in the compromised (close or positive) margins, are depicted in Table 2. Compared to the previous series published by our group (*n* = 142), deep margin evaluability significantly rose from 62.7 to 98.0% (*p* < 0.001) (15). Both cohorts were comparable concerning cT distribution (χ^2^, *p* = 0.271) but showed a significant difference in the distribution of primary and salvage cases, with the historical cohort consisting of 34/142 (23.8%) salvage cases compared to 11/104 (10.6%) salvage cases in the current cohort (χ^2^, *p* = 0.008) (Table 3). However, when primary TLM subpopulations were compared between both the historic (*n* = 109) and current (*n* = 93) cohorts, the significant rise in deep margin evaluability was again confirmed, evolving from 60.6% in the historic primary TLM cohort to 97.7% in the current primary TLM cohort (*p* < 0.001), in the absence of significant differences in cT distribution (χ^2^, *p* = 0.397). In general, 18 second look procedures were performed, yielding residual invasive SCC in 5 cases (27.8%). According to our institutional policy, most patients with a positive deep margin were scheduled for a second look TLM (*n* = 9 out of 12 patients with a positive deep margin; 75.0%); one patient was submitted to close follow-up and for two patients, definitive radiotherapy was preferred above second look TLM because of deep and multiple superficial margin positivity in combination with the surgeon's estimate of a low probability of achieving free margins in a second look procedure. In nine patients who underwent a second look procedure because of deep margin positivity, invasive SCC was found in four specimens (44.4%; three primary cases and one salvage case) with the resection considered adequate and the patients submitted to postoperative follow-up in three cases. One patient was eventually scheduled for definitive radiotherapy because margins were again considered compromised. This finding is in contrast with our previous study, in which we reported 28 second look TLM-procedures performed in 142 patients (19.7%) because of a compromised deep margin but without a single second look TLM-procedure yielding any residual malignancy (15). As a consequence, the “true positive rate” of the deep margins significantly rose from 0% toward 44.4% after introducing the new TLM specimen processing protocol (Fisher's exact test, *p* = 0.002). Figure 3 illustrates the excellent evaluability of the deep margin on a microscopic view of a transverse section of a TLM specimen—pig liver carrier complex. In addition to the 9 second look procedures performed for deep margin positivity, 9 second look TLM's were scheduled for (multiple) superficial margin positivity. In 1 (primary) case, residual invasive SCC was found in the second look specimen, which results in a “true positive rate” of the superficial margins of 11.1%. However, the “true positive rate” of deep (44.4%) and superficial margins (11.1%) did not prove significantly different (Fisher's exact, *p* = 0.294).

**Table 1:** Overview of margin evaluability and margin status according to location of the margin (deep, cranial, caudal, posterior tip, and anterior tip).

	**Deep margin *n*, (%)**	**Cranial margin *n*, (%)**	**Caudal margin *n*, (%)**	**Anterior tip *n*, (%)**	**Posterior tip *n*, (%)**
Free	66 (66.7)	44 (43.6)	46 (45.5)	31 (31)	32 (31.7)
close	19 (19.2)	16 (15.8)	21 (20.8)	7 (7)	5 (5.0)
positive	12 (12.1)	27 (26.7)	21 (20.8)	43 (43)	44 (43.6)
Non-evaluable	1.9 (2.0)	14 (13.9)	13 (12.9)	19 (19)	20 (19.8)

**Table 2:** Overview of histologic specifications (invasive SCC, CIS, dysplasia) of the compromised (close or positive) margins.

	**Deep margin *n*, (%)**	**Cranial margin *n*, (%)**	**Caudal margin *n*, (%)**	**Anterior tip *n*, (%)**	**Posterior tip *n*, (%)**
SCC	31 (100)	12 (29.3)	12 (29.3)	16 (34.8)	12 (25.0)
CIS	-	8 (19.5)	10 (24.4)	8 (17.4)	14 (29.2)
Dysplasia	-	21 (51.2)	19 (46.3)	22 (47.8)	22 (45.8)

*CIS, carcinoma in situ; SCC, squamous cell carcinoma*.

**Table 3:** Comparison of the historical TLM cohort and the current TLM cohort with regards to cT distribution and proportion primary vs. salvage TLM cases.

		**Historic cohort (*n* = 142), *n* (%)**	**Current cohort (*n* = 104), *n* (%)**	***p*-value (χ^2^)**
cT				0.0271
	cTIS	0 (0)	1 (1)	
	cT1a	89 (62.2)	68 (65.4)	
	cT1b	12 (8.4)	14 (13.5)	
	cT2	41 (28.7)	20 (19.2)	
	cT3	1 (0.7)	1 (1)	
Setting				**0.008**
	Primary	109 (76.2)	93 (89.4)	
	Salvage	34 (23.8)	11 (10.6%)	

*Bold = statistically significant difference*.

## Discussion

Margin assessment of TLM specimens is generally known to be a serious challenge for pathologists. The combination of piecemeal resections, specimen orientation issues, specimen shrinkage, laser coagulation artifacts at the level of the margins on one hand and the ultra-narrow surgical margins in order to preserve as much healthy laryngeal tissue (and laryngeal function) as possible on the other hand, imply difficult and often inaccurate evaluation of the margins. In relation to this, high rates of non-evaluable or indeterminate margins after TLM have been reported, ranging from 17.2 to 33% (16). After having been confronted ourselves with substantial rates of non-evaluable margins (37.3%) upon retrospective review of a historic TLM cohort in our center, our group searched for ways to optimize specimen orientation and evaluation in order to reduce the rate of indeterminate margins (15). A commonly used way to orient TLM specimens is inking the margins and pinning the specimen on cardboard. However, this technique lacks standardization concerning orientation designation, is susceptible to disintegration of the specimen-cardboard complex during transport, and, most important, the specimen needs to be removed from the cardboard prior to specimen processing in the pathology lab, potentially resulting in permanent loss of details concerning orientation during the further processing and specimen evaluation. This is particularly an issue when dealing with piecemeal resected specimens, where the transected part of the tumor holds the danger of being erroneously judged as “positive margin,” leading—by default—to negative second look procedures.

Inspired by earlier research in which an innovative way of orienting and fixing laryngeal specimens on dehydrated cucumber was described, but following the observation of specimen loosening in the microtome when using dehydrated cucumber, we developed a new protocol of orienting, inking and fixing TLM specimens on pig liver slices (17, 18). This study prospectively evaluated feasibility of this new technique in daily practice and aimed at identifying its potential added value on margin status reporting.

Concerning the evaluation of feasibility, introduction of the new work-up protocol resulted in a substantial time investment by the surgeon who orients, inks and fixes the specimen in the operating theater (mean duration: 5.1 min) and the pathology lab technician, who prepares the specimen-carrier complex for slicing (mean duration 8.9 min). However, as the specimen manipulation by the surgeon is performed immediately after finishing the TLM procedure, it does not result in a prolonged surgical time, neither does it influence consecutive patient turnover in the OR. Moreover, in centers where the distance between the operating room and pathology lab is substantial, introduction of this protocol could be more time efficient for the surgeon when compared to the common scenario of the surgeon joining the pathologist for specimen orientation in the lab (the hitherto proposed “ideal scenario” that proves rarely possible from a practical point of view). In addition, one must be aware that these durations are “absolute” durations, not representing the extra time investment when compared to the “old standard.” Especially for the lab technician, preparation of the carrier-specimen complex is actually not substantially different from the classic specimen preparation. As such, the time investment related to the introduction of this new protocol stays well within the limits of what is perceived as feasible and reasonable, as well by surgeons and pathologists. Apart from this objective feasibility analysis, analysis of subjective parameters confirmed overall feasibility: in the operating room, no difficulties in orientation nor fixation were reported in 87.5 and 88.2% of specimens, respectively, and in the lab, the pathologist did not encounter any difficulties with specimen orientation in 89.4% of specimens. Moreover, the accompanying photographs were judged helpful during orientation and further processing in 84.6% of specimens. Substantial difficulties during lab processing and pathologic examination were reported in 17.7% of specimens, the most common problems being the presence of laser coagulation artifacts hampering margin evaluation and problems related to the pig liver carrier with specimen release during processing.

An important objective of the introduction of this new specimen work-up protocol and its prospective evaluation, was achieving a reduction in the high rate of margins judged as “non-evaluable” by the pathologist. Compared to our historical cohort (*n* = 142), deep margin evaluability significantly rose from 62.7 to 98.0% (*p* < 0.001) and proved independent from treatment setting, with 2.3 vs. 0% non-evaluable margins in the primary TLM and salvage TLM subgroups, respectively (*p* = 0.717) (15). However, we still observed a 12.1% rate of positive deep margins (*n* = 12). As a previous multicenter study confirmed positive deep margins to be associated with a higher local recurrence risk, a slight but significant reduction in disease specific survival (DSS) and lower probability of organ preservation, positive deep margin status remains in our center an important indication for performing a second look TLM procedure with additional resection of the initial wound bed (19). However, based on this philosophy, in our historical cohort 28 (19.7%) patients were scheduled for a second look TLM-procedure; yet not a single second look TLM-procedure yielded residual malignancy, suggesting a high rate of false positive section margins (0% true positive rate) (15). This finding is supported by data from Ansarin, who reported persistence of disease in the resection specimens obtained by second-look TLM because of positive or close margins or dysplasia at margins in only 6 out of 90 patients (6.6% true positive rate) (4). After introduction of this new pathology work-up protocol, we observed a significant rise of the “true positive rate” of the deep margins toward 44.4% (*p* = 0.002), with second look TLM procedures now yielding residual invasive SCC in four out of nine patients. In contrast, it seems that, even with the introduction of this protocol, a reliable evaluation of superficial margins continued to be difficult, especially with regards to the anterior, and posterior mucosal tips. This was illustrated by the significantly higher rates of non-evaluability of the superficial margins and by the significantly higher rates of positive (for invasive SCC, CIS, and dysplasia) superficial margins when compared to the deep margins. Given the fact that during TLM, adequate superficial margins are delineated using highly magnified microscopic view (which is even more obvious than determining the deep margin), a high rate of false positive superficial margins is suspected. This suspicion is additionally supported by the observation that out of 9 second look TLM's performed for (multiple) superficial margin positivity, only 1 second look yielded a specimen with residual invasive SCC (11.1%). It is likely that the difficult assessment of the superficial mucosal margins, when compared to the deep margin assessment, is related to more pronounced laser coagulation and crushing artifacts, more detrimental effect of the tissue shrinkage and more loosening of the carrier-specimen complex at the mucosal margins with subsequent curling of the epithelium and lamina propria at the specimen's edges, all making evaluation more challenging. Moreover, due to trimming of the paraffin block during processing, a minimum of tissue is lost at the level of the anterior and posterior mucosal tips, which could be an additional reason for the high rate of compromised margins at the tips. Based on these findings, as well as on previously published results, we favor a pragmatic approach concerning positive mucosal margins, advocating a second look procedure for these patients with multiple mucosal margins positive for invasive SCC or CIS (9, 19). In our opinion, apart from the margin status on pathologic examination, the intra-operative opinion of the experienced surgeon on resection radicality, especially for the superficial margins, continues to be an important factor in decision-making concerning second-look procedures or adjuvant therapy. Related to this, it has been shown that good laryngeal exposure is an important factor in obtaining safe margins (20). With regards to true positive superficial margins, the intra-operative use of rigid endoscopy with narrow-band imaging (NBI) has been suggested as a useful adjunct in achieving an optimal superficial margin delineation (beyond the zones of CIS and dysplasia) and has been shown to potentially decrease the rate of (true) positive superficial margins (21). Moreover, the use of flexible endoscopy with NBI in the office-setting developed a proven track record in optimizing detection rate of persistent or recurrent disease after TLM and has as such been suggested as a better post-TLM follow-up modality when compared to white light endoscopy (22). This could be an additional reason to evolve to a less aggressive attitude concerning second look procedures in case of doubtful or positive superficial margins, opting for a close follow-up with NBI-endoscopy instead. As pathologic interpretation of TLM specimens continues to be complicated, intraoperative frozen section analysis has been suggested as a potential adjunct in margin assessment, aiming at a reduction of the high rate of false positive margins and, relates to this, reducing the need for second look procedures (11). On one hand, the correlation with permanent histology examination proves to be high (up to 94%) (11). On the other hand, obtaining frozen sections damages the specimen being prepared for definitive histopathological examination, it requires extra operating time and frozen sections are unrepresentative of the whole mucosal margins (4). Additionally, Fang et al. found that the status of the initial frozen-section and not the definitive frozen-section of the margin was a robust predictor of overall survival and early recurrence: if the surgeon was not able to obtain a negative margin by frozen section analysis initially, the patient was more likely to experience recurrence of disease within the first year, regardless of achieving clear margins by the end of resection, thereby questioning its real clinical value (23). Wound bed biopsies after the initial resection is advocated by some authors as an alternative to intra-operative frozen sections, as it can predict local recurrence independent from margin status of the specimen, guiding further treatment. However, sampling errors leading to false negative results need to be taken into account (24).

We identified various advantages related to the introduction of this new work-up protocol of TLM specimens. First, it is cheap, quick to learn and no specific equipment is necessary. Second, this protocol could be more practical and time-efficient than orienting the specimen together with the pathologist, especially in large hospitals with a considerable distance between the OR and the pathology lab. Third, with this protocol, a standardized way of processing the TLM specimen from the moment it leaves the patient till the moment it is actually evaluated under the microscope, has been introduced, leading to more accuracy in margin analysis and uniform and easy to interpret pathology reports. Together with a significantly improved reliability of the deep margin status and a significant decline in the portion of non-evaluable or indeterminate margins, this resulted in an increase in the surgeon's confidence in the pathologic assessment, facilitating a better and easier postoperative decision-making process with regards to second-look procedures, adjuvant treatment and follow-up intensity. As false positive margins after TLM may result in overtreatment (e.g., unnecessary second-look TLM procedures, adjuvant radiotherapy), the reduction in false positive and non-evaluable or indeterminate margins could potentially result in a reduction in overtreatment.

Disadvantages of the new pathology protocol are scarce and are mainly limited to practical problems during specimen processing (e.g., specimen release from the pig liver carrier; in this respect being parsimonious with the cyanoacrylate glue is important) and pathologic analysis (e.g., laser coagulation artifacts hampering margin evaluation). As this prospective study was designed as a feasibility study, it is at this moment not clear whether the introduction of this new protocol results in superior oncological outcomes such as a decrease in local recurrence rate, a rise in DSS and OS or a rise in organ preservation rate. A *post-hoc* analysis after a further follow-up interval (mean, 2 years) is underway to answer this question. It is possible that the enhanced margin status reliability offered by this pathology work-up protocol reduces the amount of false negative margins and as such reduces the chance of submitting a patient with true positive margins to follow-up instead of performing a second look TLM procedure. As prior reports showed a significant positive prognostic effect of negative margins on overall survival and local control, regardless of the number of procedures required to obtain these free margins, we would expect better oncologic outcomes in this patient group when compared to the historic cohort (25).

## Conclusion

The new and standardized technique of oriented fixation of TLM specimens on a pig liver carrier proves feasible both in the OR and lab setting and results in high margin evaluability rates, especially for the deep margin, as well as a decreased rate of false positive deep margins.

## Synopsis

We developed a new standardized technique of oriented fixation of transoral laser microsurgery (TLM) specimens on pig-liver slices which proves to be feasible both in the operating room and lab setting and results in high margin evaluability rates, especially for the deep margin, as well as a decreased rate of false positive deep margins when compared to a historical TLM cohort.

## Data Availability Statement

The raw data supporting the conclusions of this article will be made available by the authors, without undue reservation.

## Ethics Statement

The studies involving human participants were reviewed and approved by University Hospital Leuven Committee for Medical Ethics. The patients/participants provided their written informed consent to participate in this study.

## Author's Note

The abstract of this paper was accepted as a podium presentation during the European Laryngological Society (ELS) meeting in Stuttgart, Germany (initially scheduled 17–20 June 2020, but postponed due to COVID-19 pandemic).

## Author Contributions

JM and VV contributed to study set-up, data collection, data quality control, data analysis (statistics), drafting manuscript, and review of manuscript. EH contributed to study set-up, data collection, drafting manuscript, and review of manuscript. SP contributed to data collection, drafting manuscript, and review of manuscript. SN and PD contributed to drafting manuscript and review of manuscript. All authors contributed to the article and approved the submitted version.

## Conflict of Interest

The authors declare that the research was conducted in the absence of any commercial or financial relationships that could be construed as a potential conflict of interest.

## References

[1] Hartl DM Ferlito A Brasnu DF Langendijk JA Rinaldo A Silver CE. Evidence-based review of treatment options for patients with glottic cancer. Head Neck. (2011) 33:1638–48. 10.1002/hed.21528 21990228

[2] Ambrosch P. The role of laser microsurgery in the treatment of laryngeal cancer. Curr Opin Otolaryngol Head Neck Surg. (2007) 15:82–8. 10.1097/MOO.0b013e3280147336 17413407

[3] Peretti G Piazza C Cocco D De Benedetto L Del Bon F Redaelli De Zinis LO. Transoral CO^2^ laser treatment for Tis-T3 glottic cancer: The University of Brescia experience on 595 patients. Head Neck. (2010) 32:977–83. 10.1002/hed.21278 19902535

[4] Ansarin M Cattaneo A De Benedetto L Zorzi S Lombardi F Alterio D. Retrospective analysis of factors influencing oncologic outcome in 590 patients with early-intermediate glottic cancer treated by transoral laser microsurgery. Head Neck. (2017) 39:71–81. 10.1002/hed.24534 27453475

[5] Feng Y Wang B Wen S. Laser surgery versus radiotherapy for T1-T2N0 glottic cancer: a meta-analysis. ORL. (2011) 73:336–42. 10.1159/000327097 22005723

[6] Mantsopoulos K Psychogios G Koch M Zenk J Waldfahrer F Iro H. Comparison of different surgical approaches in T2 glottic cancer. Head Neck. (2012) 34:73–7. 10.1002/hed.21687 21374754

[7] Karatzanis AD Psychogios G Zenk J Waldfahrer F Hornung J Velegrakis GA. Comparison among different available surgical approaches in T1 glottic cancer. Laryngoscope. (2009) 119:1704–8. 10.1002/lary.20537 19572396

[8] Meulemans J Delaere P Nuyts S Clement PM Hermans R Vander Poorten V. Salvage transoral laser microsurgery for radiorecurrent laryngeal cancer: indications, limits, and outcomes. Curr Otorhinolaryngol Rep. (2017) 5:83–91. 10.1007/s40136-017-0143-7 28367362 PMC5357496

[9] Fiz I Koelmel JC Sittel C. Nature and role of surgical margins in transoral laser microsurgery for early and intermediate glottic cancer. Curr Opin Otolaryngol Head Neck Surg. (2018) 26:78–83. 10.1097/MOO.0000000000000446 29373328

[10] Canis M Ihler F Martin A Matthias C Steiner W. Transoral laser microsurgery for T1a glottic cancer: review of 404 cases. Head Neck. (2015) 37:889–95. 10.1002/hed.23688 24623709

[11] Remacle M Matar N Delos M Nollevaux MC Jamart J Lawson G. Is frozen section reliable in transoral CO2 laser-assisted cordectomies? Eur Arch Oto-Rhino-Laryngol. (2010) 267:397–400. 10.1007/s00405-009-1101-x 19784844

[12] Meulemans J Jans A Vermeulen K Vandommele J Delaere P Vander Poorten V. Evone® flow-controlled ventilation during upper airway surgery: a clinical feasibility study and safety assessment. Front Surg. (2020) 7:6. 10.3389/fsurg.2020.00006 32185179 PMC7058692

[13] Remacle M Eckel HE Antonelli A Brasnu D Chevalier D Friedrich G. Endoscopic cordectomy. A proposal for a classification by the Working Committee, European Laryngological Society. Eur Arch Otorhinolaryngol. (2000) 257:227–31. 10.1007/s004050050228 10867840

[14] Remacle M Van Haverbeke C Eckel H Bradley P Chevalier D Djukic V. Proposal for revision of the European Laryngological Society classification of endoscopic cordectomies. Eur Arch Otorhinolaryngol. (2007) 264:499–504. 10.1007/s00405-007-0279-z 17377801

[15] Meulemans J Bijnens J Delaere P Vander Poorten V. Up-front and salvage transoral laser microsurgery for early glottic squamous cell carcinoma: a single centre retrospective case series. Front Oncol. (2018). 8:186. 10.3389/fonc.2018.00186 29892574 PMC5985398

[16] Jumaily M Faraji F Osazuwa-Peters N Walker RJ Ward GM. Prognostic significance of surgical margins after transoral laser microsurgery for early-stage glottic squamous cell carcinoma. Oral Oncol. (2019) 97:105–11. 10.1016/j.oraloncology.2019.08.005 31473467

[17] Murray CE Cooper L Handa KK MacLeod T MacKenzie K. A technique for the orientation of endoscopically resected laryngeal lesions. Clin Otolaryngol. (2007) 32:201–3. 10.1111/j.1365-2273.2007.01337.x 17550514

[18] Robertson S Cooper L McPhaden A MacKenzie K. Refining the “cucumber” technique for laryngeal biopsy. J Laryngol Otol. (2011) 125:626–9. 10.1017/S0022215111000119 21371370

[19] Fiz I Mazzola F Fiz F Marchi F Filauro M Paderno A. Impact of close and positive margins in transoral laser microsurgery for Tis–T2 glottic cancer. Front Oncol. (2017) 7:1–9. 10.3389/fonc.2017.00245 29085805 PMC5650697

[20] Piazza C Paderno A Grazioli P Del Bon F Montalto N Perotti P. Laryngeal exposure and margin status in glottic cancer treated by transoral laser microsurgery. Laryngoscope. (2018) 128:1146–51. 10.1002/lary.26861 28895157

[21] Garofolo S Piazza C Del Bon F Mangili S Guastini L Mora F. Intraoperative narrow band imaging better delineates superficial resection margins during transoral laser microsurgery for early glottic cancer. Ann Otol Rhinol Laryngol. (2015) 124:294–8. 10.1177/0003489414556082 25358609

[22] Piazza C Cocco D De Benedetto L Del Bon F Nicolai P Peretti G. Narrow band imaging and high definition television in the assessment of laryngeal cancer : a prospective study on 279 patients. Eur Arch Otorhinolaryngol. (2010) 267:409–14. 10.1007/s00405-009-1121-6 19826829

[23] Fang TJ Courey MS Liao CT Yen TC Li HY. Frozen margin analysis as a prognosis predictor in early glottic cancer by laser cordectomy. Laryngoscope. (2013) 123:1490–5. 10.1002/lary.23875 23401100

[24] Hendriksma M Montagne MW Langeveld TPM Veselic M van Benthem PPG Sjögren EV. Evaluation of surgical margin status in patients with early glottic cancer (Tis-T2) treated with transoral CO2 laser microsurgery, on local control. Eur Arch Oto-Rhino-Laryngology. (2018) 275:2333–40. 10.1007/s00405-018-5070-9 30027440 PMC6096566

[25] Karatzanis AD Waldfahrer F Psychogios G Hornung J Zenk J Velegrakis GA. Effect of repeated laser microsurgical operations on laryngeal cancer prognosis. Head Neck. (2010) 32:921–8. 10.1002/hed.21272 19924806

